# Fast and robust deconvolution of tumor infiltrating lymphocyte from expression profiles using least trimmed squares

**DOI:** 10.1371/journal.pcbi.1006976

**Published:** 2019-05-06

**Authors:** Yuning Hao, Ming Yan, Blake R. Heath, Yu L. Lei, Yuying Xie

**Affiliations:** 1 Department of Statistics and Probability, Michigan State University, East Lansing, United States of America; 2 Department of Computational Mathematics, Science and Engineering, Michigan State University, East Lansing, United States of America; 3 Department of Mathematics, Michigan State University, East Lansing, United States of America; 4 Department of Periodontics and Oral Medicine, University of Michigan School of Dentistry Ann Arbor, United States of America; 5 University of Michigan Rogel Cancer Center, Ann Arbor, United States of America; Ottawa University, CANADA

## Abstract

Gene-expression deconvolution is used to quantify different types of cells in a mixed population. It provides a highly promising solution to rapidly characterize the tumor-infiltrating immune landscape and identify cold cancers. However, a major challenge is that gene-expression data are frequently contaminated by many outliers that decrease the estimation accuracy. Thus, it is imperative to develop a robust deconvolution method that automatically decontaminates data by reliably detecting and removing outliers. We developed a new machine learning tool, Fast And Robust DEconvolution of Expression Profiles (FARDEEP), to enumerate immune cell subsets from whole tumor tissue samples. To reduce noise in the tumor gene expression datasets, FARDEEP utilizes an adaptive least trimmed square to automatically detect and remove outliers before estimating the cell compositions. We show that FARDEEP is less susceptible to outliers and returns a better estimation of coefficients than the existing methods with both numerical simulations and real datasets. FARDEEP provides an estimate related to the absolute quantity of each immune cell subset in addition to relative percentages. Hence, FARDEEP represents a novel robust algorithm to complement the existing toolkit for the characterization of tissue-infiltrating immune cell landscape. The source code for FARDEEP is implemented in R and available for download at https://github.com/YuningHao/FARDEEP.git.

## Author summary

Rapidly emerging evidence suggests that the tumor immune microenvironment not only predisposes cancer patients to diverse treatment outcomes but also represents a promising source of biomarkers for better patient stratification. Different from the immunohistochemistry-based scoring practice, which focuses on a few selected marker proteins, immune deconvolution pipelines inform a previously untapped method to comprehensively reveal the tumor-infiltrating immune landscape. Recognizing the numerous strengths of existing immune deconvolution algorithms, here we show data outliers, which are inevitable in whole tissue sequencing data sets, substantially skew estimation results. Moreover, an estimate related to the absolute amount of each immune subset offers valuable insight into the nature of the host response in addition to percentage information alone. Thus, we engineered a new immune deconvolution pipeline, coined as Fast and Robust Deconvolution of Expression Profiles (FARDEEP), to automatically detect and remove outliers prior feeding data into the deconvolution algorithm and to provide estimates related to the absolute quantity of each immune subset. Utilizing both synthetic and real data sets, we found that FARDEEP returns superior coefficients and offers a robust tool to reveal the immune landscape of human cancers.

## Introduction

Immune checkpoint blockade has revolutionized the rational design of neoadjuvant cancer therapies. Compelling evidence suggests that a favorable tumor immune microenvironment underpins better clinical responses to radiotherapy, chemotherapy, and immunotherapy [1–3]. Immunohistochemistry (IHC)-based immunoscores, which quantify the number of CD8^+^ cytotoxic T lymphocytes and CD45RO^+^ memory T cells, show better prognostic potential than conventional pathological methods in colon cancer patients [4, 5]. Hence, harnessing the composition of intra-tumoral immune cell infiltration is a highly promising approach to stratify tumors [6–11]. The current IHC immunoscoring approach has two limitations. First, the interpretation of immune cell subsets varies among pathologists and institutions, thus lacking a consistent standard for the scoring practice. Second, only a limited number of biomarkers can be assessed simultaneously, which prevents a comprehensive annotation of the immune contexture in the tumor microenvironment (TME). Hence, robust methods for genome data-informed cell type quantitation are in urgent need.

Immunogenomics presents an unprecedented opportunity to resolve the intra-tumoral immune landscape. Cell type deconvolution using leukocyte signature gene expression profiling is a highly promising approach to estimate the global immune cell composition from whole tumor gene expression data [12–17]. However, a significant technical obstacle is that the efficacy and accuracy of gene expression deconvolution are limited by the large number of outliers, which are frequently observed in tumor gene expression datasets [18]. The first step towards enhancing the overall gene deconvolution algorithms is to improve methods for outliers identification and processing. Those outliers include genes with abnormal expression value which may be caused by measurement error, environmental effect, expression from non-immune cells, or natural fluctuations in certain type of immune cells. Notably, the current immune deconvolution gene signature matrix relies on the profiling of differentially expressed genes among different immune subsets. Frequent contamination of transcripts reading from cancer cells may significantly bias the algorithms. In this study, we report a novel FAst and Robust DEconvolution of Expression Profiles (FARDEEP) method that significantly improves the estimation of coefficients.

Let *y*_*i*_ be the observed expression value for the *i*th gene; ***x***_***i***_, a *p*-dimensional vector, be the expected expression of the *i*th gene for the *p* different cell types; and ***X*** = [***x***_1_, …, ***x***_*n*_]′ be the signature matrix. The gene-expression deconvolution problem can be formulated as follows,

$$\begin{array}{c} y_{i}=x_{i}^{\prime }\beta +\varepsilon_{i}, \end{array}$$ (0.1)

where $\beta \in R^{p}$ is an unknown parameter corresponding to the compositions of *p* cell types, and *ε*_*i*_ is a noise term with a mean of 0. Several methods were proposed to solve this deconvolution problem. To enforce the non-negativity of ***β*** in (0.1), several algorithms, such as the Non-Negative Least Square (NNLS), Non-negative Maximum Likelihood (NNML) frameworks and the perturbation model (PERT) were developed. They all rely on the signature matrix (*X*) derived from Microarray experiments [14, 19–24]. To extend this work to RNA-seq data, Finotello *et al*. [14] proposed a constraint linear model with a signature matrix derived from RNA-seq data. Additionally, the gene expression of each cell may vary depending on its microenvironment and other factors, which will lead to a biased estimation. To address this issue, Microarray Microdissection with Analysis of Differences (MMAD) incorporates the concept of the effective RNA fraction and estimates coefficients using a maximum likelihood approach [25]. To further adapt deconvolution to high-dimensional settings, Altboum *et al*. [26] proposed a penalized regression framework, Digital Cell Quantifier (DCQ), to encourage sparsity for the estimated ***β*** using the elastic net [27]. Cell-type identification by estimating relative subsets of RNA transcripts (CIBERSORT) uses *ν*-support vector regression (*ν*-SVR) to enhance the robustness of gene expression deconvolution. CIBERSORT performs a regression by finding a hyperplane that fits as many data points as possible within a tube whose vertical length is a constant *ε* [12]. The *ε*-tube provides a region in which estimation errors are ignored. This model does not include an intercept to capture contributions of other contents. Additionally, to increase the computational efficiency, CIBERSORT applies Z-normalization to the data before fitting the regression, which may introduce estimation bias. Based on the CIBERSORT framework, several extensions have been proposed to overcome limitations such as platform inconsistency between signature and mixture matrices and low estimation accuracy for *γδ* T cell [15–17]. However, the quantitative information of cell proportions of these two approaches is built on CIBERSORT whose performance may be challenged by frequent outliers in whole tumor tissue transcriptomes. To reduce the dependence on the signature matrix, xCell utilizes the concept of single-sample gene set enrichment analysis (ssGSEA) to calculate an immune cell score which could predict the enrichment of immune cells [13]. Despite its robustness, xCell relies much on the ranking of gene expression value which leads to suboptimal solution for the estimation accuracy. Overall, a robust method that determines both the distribution and absolute volume of tumor-infiltrating lymphocytes (TILs) will further improve the current gene deconvolution pipeline.

To handle the heavily contaminated gene expression data and provide absolute cell abundance estimation, we developed a robust method based on the Least Trimmed Square (LTS) framework [28, 29]. LTS finds *h* observations with smallest residuals, and the estimator $\hat{\beta }$ is the least squares fit over these *h* observations. LTS is an NP-hard problem, and Rousseeuw and Driessen [30] proposed a stochastic FAST-LTS algorithm. Nevertheless, it may give a suboptimal fitting result and get much slower when the sample size and dimension of variables become larger and higher since its accuracy relies on the initial random *h*-subsets and the number of initial subsets. When *n* is the sample size and *p* is the number of coefficients, *h* is suggested to be the smallest integer that is not less than (*n* + *p* + 1)/2 to remove as many outliers as possible while keeping an unbias result. Using the information of only half of the data reduces the power of the estimator because the amount of outliers in the real case cannot be presumed and can be small. Xu *et al*. [31] proposed an adaptive least trimmed square which is not limited to the randomness of initial subset but only applied the binary dataset. In this study, we extend the adaptive least trimmed square to introduce a model-free method, which could find the number of outliers automatically based on LTS. FARDEEP provides a flexible framework which is suitable for both Microarray and RNA-seq data using LM22 and Immunostate signature matrices respectively. As evidence of high fidelity and robustness, FARDEEP exhibits superior performance in simulated and real-world datasets.

## Materials and methods

### Model formulation

The usual linear deconvolution model can be expressed as below,

$$\begin{array}{c} y=X\beta +\varepsilon , \end{array}$$

where $y\in R^{n}$ is the observed expression data for *n* immune subset signature genes, $X\in R^{n\times p}$ denotes a mean gene expression signature matrix for *p* different cell types, $\beta \in R^{p}$ contains each unknown cell type abundance, and $\varepsilon \in R^{n}$ is a vector of random errors with zero mean and variance of *σ*^2^***I***. To incorporate outliers, we propose the following model

$$\begin{array}{c} y=X\beta +\tau +\varepsilon , \end{array}$$ (0.2)

where parameter ***τ*** = (*τ*_1_, …, *τ*_*n*_)′ is a sparse vector in $R^{n}$ with *τ*_*i*_ ≠ 0 indicating the *i*th gene is an outlier.

Under the formulation of (0.2), let ${\hat{\beta }}_{\text{ols}}={\left(X^{⊤}X\right)}^{-1}X^{⊤}y$ be the Ordinary Least Square (OLS) estimate and ***H*** = ***X***(***X***^⊤^***X***)^−1^***X***^⊤^ be the projection matrix. The residuals ***r*** = (*r*_1_, …, *r*_*n*_) using OLS could be formulated as

$$\begin{array}{c} r=y-{X\hat{\beta }}_{\text{ols}}=\left(I-H\right)\tau +\left(I-H\right)\epsilon . \end{array}$$ (0.3)

with mean of (***I*** − ***H***)***τ*** and variance of *σ*^2^(***I*** − ***H***).

### Adaptive least trimmed square

From (0.3), the residuals, *r*_*i*_ with the corresponding *τ*_*i*_ ≠ 0, would deviate from zero, which suggests that the set of outliers can be identified through thresholding as follows

$$\begin{array}{c} E=\{i:|r_{i}|>k\times r_{\text{med}}\}, \end{array}$$ (0.4)

where *E* is the set of detected outliers, *k* is a tuning parameter controlling the sensitivity of the model, and *r*_med_ is the median of ${\left\{|r\right|}_{i}{\}}_{i=1}^{n}$. We denote the number of elements in set *E* as |*E*| and let *N* be the number of true outliers in the data. First, we can use least squares and formula (0.4) to obtain a rough estimate of *E* denoted as $\hat{E}$. Let the cardinality of $\hat{E}$ be $\bar{N}$. Since the model at this moment is inaccurate with contamination of outliers, $\bar{N}$ is an overestimation of *N* which can be used to get an underestimate via $\underline{N}=\alpha_{1}\bar{N}$ with *α*_1_ ∈ (0, 1). With $\underline{N}$, we can then update the least square fitting after removing the $\underline{N}$ samples with the largest absolute value of residuals and obtain an improved estimate of *E* and the corresponding $\bar{N}$. We can improve the model by repeating the procedure, but we need to increase the underestimate of outliers, $\underline{N}$, by a factor of *α*_2_ with *α*_2_ > 1 for each iteration to force the convergence between $\bar{N}$ and $\underline{N}$. In sum, we initialize our algorithm by setting

$$\begin{array}{cc} {\hat{\beta }}^{\left(0\right)} & ={\left(X^{⊤}X\right)}^{-1}X^{⊤}y,\\ r^{\left(0\right)} & =y-X{\hat{\beta }}^{\left(0\right)}, \end{array}$$

which is the OLS solution. For the *j*th iteration, where *j* ≥ 1, we update ${\bar{N}}^{\left(j\right)}$ by

$$\begin{array}{c} {\bar{N}}^{\left(j\right)}=\{\begin{array}{cc} |\{i:|r_{i}^{\left(j-1\right)}|>r_{\text{med}}^{\left(j-1\right)}\}|, & j=1,\\ \text{min}(|\{i:|r_{i}^{\left(j-1\right)}|>k\cdot r_{\text{med}}^{\left(j-1\right)}\}|,\;{\bar{N}}^{\left(j-1\right)}), & j\geq 2. \end{array} \end{array}$$ (0.5)

where the min(⋅, ⋅) operator guarantees that ${\bar{N}}^{\left(j\right)}$, an overestimation of *N*, is non-increasing. Similarly, we update ${\underline{N}}^{\left(j\right)}$ through

$$\begin{array}{c} {\underline{N}}^{\left(j\right)}=\{\begin{array}{cc} \lceil \alpha_{1}{\bar{N}}^{\left(j\right)}\rceil , & j=1,\\ \text{min}\{\left\lceil \alpha_{2}{\underline{N}}^{\left(j-1\right)}\right\rceil ,\;{\bar{N}}^{\left(j\right)}\}, & j\geq 2, \end{array} \end{array}$$ (0.6)

where ⌈*x*⌉ means the ceiling of x∈R, *α*_1_ ∈ (0, 1) is used to obtain a lower bound for *N* in the first step, *α*_2_ > 1 guarantees the monotonicity of ${\underline{N}}^{\left(j\right)}$, and the min(⋅, ⋅) operator guarantees ${\underline{N}}^{\left(j\right)}$ is smaller than ${\bar{N}}^{\left(j\right)}$. Then we update $\hat{\beta }$ and ***r*** after removing ${\underline{N}}^{\left(j\right)}$ outliers by

$$\begin{array}{cc} {\hat{\beta }}^{\left(j\right)} & ={\left(X^{(j)⊤}X^{\left(j\right)}\right)}^{-1}X^{(j)⊤}y^{\left(j\right)},\\ r^{\left(j\right)} & =y-X{\hat{\beta }}^{\left(j\right)}. \end{array}$$

We repeat this procedure until $\underline{N}$ and $\bar{N}$ converge.

Hence, we hereby report a novel approach, coined as adaptive Least Trimmed Square (aLTS), to automatically detect and remove contaminating outliers. Our aLTS is an extension of the iterative LTS algorithm proposed by Xu *et al*. [31] which is designed for binary output such as the comparison between two images or videos.

### FARDEEP

Because the abundance of cell types are always non-negative, we replaced the OLS regression in the aLTS procedure with non-negative least square regression (NNLS). By applying the modified aLTS to the deconvolution model (0.2) and solving the following problem,

$$\begin{array}{c} \hat{\beta }=\underset{\beta }{\text{argmin}}\;{\left\|y-X\beta \;\right\|}_{2}^{2},\;\text{subject}\;\text{to}\;\beta \geq 0 \end{array}$$

using Lawson-Hanson algorithm [19], we developed a robust tool, FARDEEP, for cellular deconvolution summarized in Algorithm 1.

One unique advantage of FARDEEP is that it is fast and guarantees to converge within finite steps, which is summarized in the following theorem.

**Algorithm 1** FAst and Robust DEconvolution of Expression Profiles

**Input**: *k* > 0, 0 < *α*_1_ < 1, *α*_2_ > 1, ***y***, ***X***

**Initialization**: solving the following NNLS problem

$$\begin{array}{cc} {\hat{\beta }}^{\left(0\right)} & =\underset{\beta }{\text{argmin}}\;{\left\|y-X\beta \right\|}_{2}^{2},\;\text{subject}\;\text{to}\;\beta \geq 0;\\ r^{\left(0\right)} & =y-{X\hat{\beta }}^{\left(0\right)}. \end{array}$$

 1: compute ${\bar{N}}^{\left(1\right)}$ and ${\underline{N}}^{\left(1\right)}$ using (0.5) and (0.6);

 2: solving the NNLS problem after removing ${\underline{N}}^{\left(1\right)}$ genes with largest residuals, and update ${\hat{\beta }}^{\left(1\right)}$, ***r***^(1)^.

 3: **repeat**

 4:  compute ${\bar{N}}^{\left(j\right)}$ and ${\underline{N}}^{\left(j\right)}$ using (0.5) and (0.6) for *j* ≥ 2;

 5:  solving the NNLS problem after removing ${\underline{N}}^{\left(j\right)}$ genes with largest residuals, and update ${\hat{\beta }}^{\left(j\right)}$, ***r***^(*j*)^;

 6: **until** $\bar{N}=\underline{N}$.

**Output**: Coefficients $\hat{\beta }$, Number of outliers $\hat{N}$, Index of outliers

**Theorem 1** Algorithm 1 (FARDEEP) stops in no more than j* steps, where

$$\begin{array}{c} j^{*}=\lfloor \frac{-\text{log}\;\alpha_{1}}{\text{log}\;\alpha_{2}}\rfloor +2. \end{array}$$

*Here* ⌊⋅⌋ *is the largest integer that is less than or equal to x*.

*Proof*. It follows from the fact that the sequence $\left\{{\bar{N}}^{\left(j\right)}\right\}$ is non-increasing, and $\left\{{\underline{N}}^{\left(j\right)}\right\}$ is a geometrically increasing sequence that is bounded by the smallest component of $\left\{{\bar{N}}^{\left(j\right)}\right\}$. Specifically, assume that j* steps have been taken in FARDEEP, then *j* has approached j* − 1, and ${\underline{N}}^{\left(j\right)}\geq \alpha_{2}{\underline{N}}^{\left(j-1\right)}$ for 0 ≤ *j* ≤ j* − 1, so

$$\begin{array}{c} {\bar{N}}^{\left(0\right)}\geq {\bar{N}}^{\left(j^{*}-2\right)}\geq {\underline{N}}^{\left(j^{*}-2\right)}\geq \alpha_{2}^{j^{*}-2}{\underline{N}}^{0}\geq \alpha_{2}^{j^{*}-2}\alpha_{1}{\bar{N}}^{0}. \end{array}$$

which leads to the result.

The $\hat{\beta }$ from FARDEEP, denoted as *TIL subset score*, is the direct estimate of the linear model without any normalization and hence reflects the absolute abundance of TILs. In addition, we can derive the relative TILs abundance from the TIL subset scores through

$$\begin{array}{c} {\tilde{\beta }}_{j}=\frac{\hat{\beta_{j}}}{\sum_{k=1}^{p}\hat{\beta_{j}}}, \end{array}$$ (0.7)

where ${\hat{\beta }}_{j}$ is the *j*th TIL subset score. In practice, the TIL subset score provides important information of patient’s tumor-infiltrating immune landscape, and we have included a discussion in S2 Text.

### Parameter tuning

There are three tuning parameters *k*, *α*_1_, and *α*_2_ in FARDEEP. Since *α*_1_ is only used in the first iteration, a relatively small *α*_1_ is preferred to ensure that FARDEEP does not remove too many outliers at the first step. In practice, FARDEEP is not sensitive to different values of *α*_1_, and *α*_2_, so we set them to 0.1 and 1.5 respectively by default. However, *k* controls the number of outliers in each iteration and is critical for the performance of FARDEEP. Thus, we tune *k* on a case-by-case basis for each sample to preserve meaningful fluctuations of gene expression levels. Effects for different tuning parameters are shown in S1 Table. Since the test group may contain outliers that influence the accuracy of the tuning result, cross-validation is not advised. Instead, we applied the Bayesian Information Criterion (BIC) and assume that the errors follow a log-normal distribution instead of a normal distribution among gene expression datasets as suggest by Beal [32]. We define the modified BIC referring to the setting of She and Owen [33]:

$$\begin{array}{c} {\text{BIC}}^{*}\left(k\right)=m\;\text{log}\frac{\sum_{i=1}^{n}1_{\left\{i\notin \hat{E}\right\}}\;{\text{log}}_{2}{\left(y_{i}-{\hat{y}}_{i}\right)}^{2}}{m}+b\left(\text{log}\left(m\right)+1\right), \end{array}$$ (0.8)

where $\hat{E}$ being the set of detected outliers, *b* is number of parameters and equals $\hat{N}+p+1$ with $\hat{N}=\left|\hat{E}\right|$ being the number of outliers, and *m* equals $n-\hat{N}$. Then, we choose the value of *k* associated with the smallest BIC*.

## Results

To test the performance of FARDEEP, we compared our approach with the existing methods using numerical simulations and real datasets. Here, we list the outlier genes detected by FARDEEP for real datasets in S4 Table. We use LM22 signature matrix containing 22 immune cell types hematopoietic cells for Microarray data and use quanTIseq signature matrix containing 10 immune cell types for RNA-Seq data. To compare the performance of different methods, we report the sum of squared error (SSE), the coefficient of determination denoted as R-squared (*R*^2^) and the Pearson correlation (*R*) defined as follows

$$\begin{array}{cc} & \text{SSE}=\sum_{j=1}^{p}{\left(\beta_{j}^{*}-{\hat{\beta }}_{j}\right)}^{2},\\ & R^{2}=1-\sum_{j=1}^{p}{\left(\beta_{j}^{*}-{\hat{\beta }}_{j}\right)}^{2}/\sum_{j=1}^{p}{\left(\beta_{j}^{*}-{\bar{\beta }}^{*}\right)}^{2},\;{\bar{\beta }}^{*}=\frac{1}{p}\sum_{j=1}^{p}\beta_{j}^{*},\\ & R=\frac{\sum_{j=1}^{p}\left(\beta_{j}^{*}-{\bar{\beta }}^{*}\right)\left({\hat{\beta }}_{j}-\bar{\hat{\beta }}\right)}{\sqrt{\sum_{j=1}^{p}{\left(\beta_{j}^{*}-{\bar{\beta }}^{*}\right)}^{2}}\sqrt{\sum_{j=1}^{p}{\left({\hat{\beta }}_{j}-\bar{\hat{\beta }}\right)}^{2}}},\;\bar{\hat{\beta }}=\frac{1}{p}\sum_{j=1}^{p}{\hat{\beta }}_{j}, \end{array}$$

where β* is the ground truth, and $\hat{\beta }$ is the estimate.

### *In silico* simulation with varied error types

To test the robustness of FARDEEP under different error conditions, we simulated three datasets refer to the setting in [33, 34] with normally distributed errors, heavy tailed errors. The observations were generated based on the linear regression model (0.2). The predictor matrix is ***X*** = (***x***_1_, …, ***x***_***n***_)′ = ***U*****Σ**^1/2^, where $U_{ij}∼U\left(0,20\right)$ and $\Sigma_{ij}=\rho^{I_{\left\{i\neq j\right\}}}$ with *ρ* = 0.5. Consider the proportion of outliers *f* ∈ {5%, 10%, 20%, 30%}, sample size *n* = 500, and number of predictors *p* = 20, we added random errors and outliers to the simulated data as follows:

- Random errors: we generated the random error vector from i) standard normal distribution, ii) *t*-distribution with 3 degrees of freedom.
- Vertical outliers: we generated a *n* dimensional zero vector ***τ*** and randomly selected *nf* elements in ***τ*** to be the outliers generated from a non-central *t*-distribution with 1 degree of freedom and a non-centrality parameter of 30.
- Leverage points: we took 20% of the contaminated data as leverage points, that is, replacing the corresponding predictors by the samples from N(2max(X),1).

The coefficients *β*_*j*_ were sampled from U(0,1), where *j* = 1, …, *p*. Based on the framework above, the dependent variable could be obtained by

$$\begin{array}{c} y=X\beta +\tau +\varepsilon . \end{array}$$

**Fig 1:**
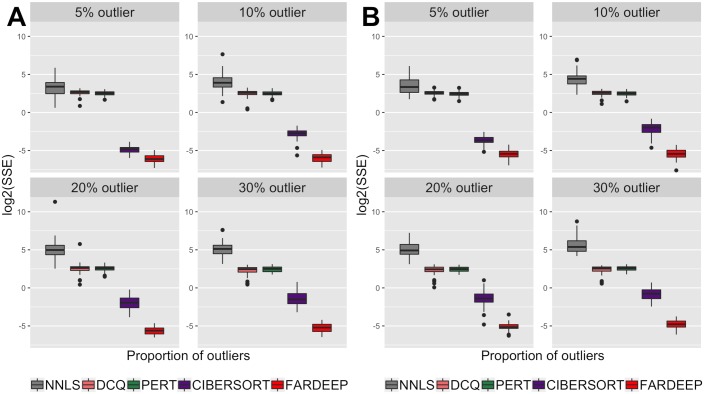
SSE of coefficients for different approaches. We simulated different percentage of outliers ({5%, 10%, 20%, 30%}) and compared the SSE for coefficients applying NNLS, DCQ, PERT, CIBERSORT, and FARDEEP. (A) random error with standard normal distribution, (B) random error with *t*-distribution.

**Fig 2:**
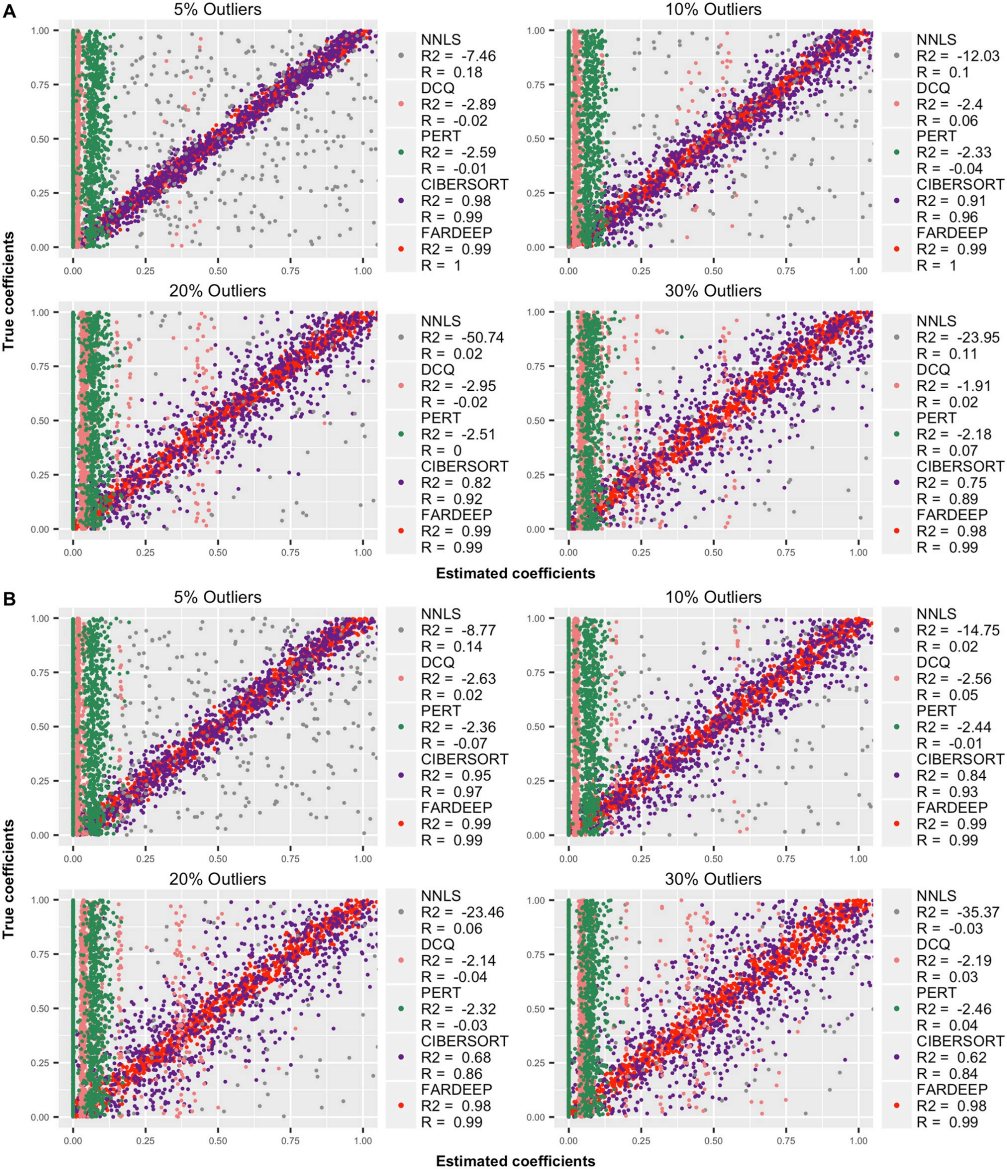
Compare the estimation accuracy of different deconvolution approaches (the values in parentheses are *R*^2^ and *R*). Based on {5%, 10%, 20%, 30%} percentage of outliers, we computed *R*^2^ to evaluate how well the estimators fit for a straight line $\hat{\beta }=\beta$. (A) random error with standard normal distribution, (B) random error with *t*-distribution.

We simulated each model 50 times. As shown in Figs 1 and 2, FARDEEP outperforms other methods, evidenced by the SSE, *R*^2^ and *R* values.

To check FARDEEP’s accuracy of outlier detection, we simulated {5%;10%;20%;30%} outliers using the same method as above for a model with both normally distributed and heavy-tailed noise. As shown in Table 1, the tuning parameter *k* decreases when the amount of outliers becomes larger, and the true positive rates always stay around 1, indicating that the tunning of k is highly effective.

**Table 1:** Tuned *k* for FARDEEP with the adjusted BIC. We simulated normally distributed errors and heavy-tailed errors respectively for different proportion of outliers and computed true positive rate and false positive rate to evaluate the tunning result.

Percentage of outliers		5%	10%	20%	30%
Normal	True positive rate	1	1	1	1
	False positive rate	0.005	0.009	0.02	0.05
	Parameter (mean of k)	3.63	2.43	1.46	1.19
Heavy-tailed	True positive rate	1	1	1	1
	False positive rate	0.05	0.05	0.08	0.11
	Parameter (mean of k)	3.03	2.06	1.35	1.14

In the supplementary material S3 Text, we also included another outlier construction scheme with ***X*** related outliers and a simulation setting with correlated responses. In both scenarios, FARDEEP dominates other methods in terms of SSE, *R*^2^ and *R* values.

### *In silico* simulation based on leukocyte gene signature matrix file

Following the similar procedure as in Newman *et al*., we randomly generated the abundance of different cells from interval [0, 1] [12]. Notably, the sum of cell abundance is not necessarily 1. The measurement errors were sampled from $2^{N(0,\;{\left(0.1\;{\text{log}}_{2}\left(s\right)\right)}^{2})}$. To incorporate outliers, we randomly selected *i*/50 of the data and replaced them with data drawn from $2^{N(10,\;{\left(0.3\;{\text{log}}_{2}\left(s\right)\right)}^{2})}$ where *i* = 1, 2, …, 25 and *s* is the standard deviation of original mixtures.

We repeated the procedure nine times and estimated the cell type abundance using FARDEEP, CIBERSORT (without converting to percentage), NNLS, PERT, and DCQ. As shown in S2 Table, we found that the SSE range for FARDEEP is 1.51 × 10^−7^ to 1.47 × 10^−4^, *R*^2^ and *R* keeps being 1 regardless of the number of outliers, while Other methods show significantly larger SSE and smaller *R*^2^, *R*.

### Synthetic dataset

**Fig 3:**
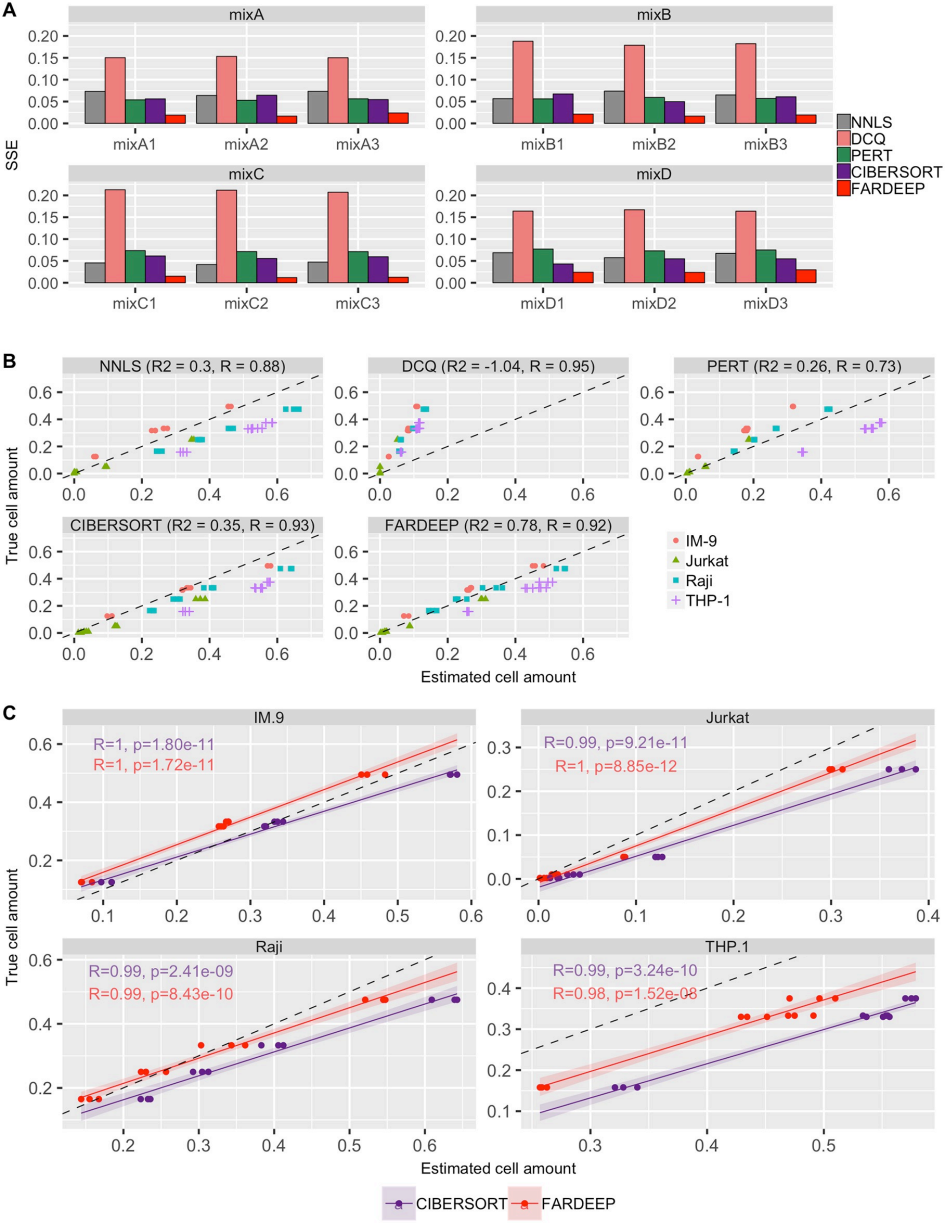
Applying different deconvolution approaches on the gene expression data of IM-9, Jurkat, Raji, THP-1 and the mixture of these four immune cell lines with known proportion (MixA, MixB, MixC, MixD). All of the mixtures were performed and measured in triplicate. (A) SSE of coefficients for FARDEEP, CIBERSORT, NNLS, PERT, DCQ. (B) The abundance of cell lines estimated from different deconvolution approaches vs. Abundance of cell lines truly mixed. The *R*^2^ and *R* values are also reported at the top of the figures. (C) Deconvolution of individual cell subsets by FARDEEP and CIBERSORT. The correlation coefficients *R*, the corresponding *p*-values against the null hypothesis of *R* = 0, trend lines with 95% confidence intervals are shown in the figures. The black dashed line represents the perfect relationship between the estimate and the true cell abundances with slope 1 and intercept 0.

We used the cell line dataset GSE11103 generated by Abbas *et al*. [35] that contains gene expression profiles of four immune cell lines (Jurkat, IM-9, Raji, and THP-1) and four mixtures (MixA, MixB, MixC, and MixD) with various ratios of cells. Before analysis, we quantile normalized the mixture data for 54675 probesets and downloaded the immune gene signature matrix with 584 probesets from CIBERSORT website. Then, we applied five deconvolution methods, including FARDEEP, CIBERSORT (without converting to percentage), DCQ, NNLS, and PERT, to calculate the sum of squared errors of the estimated abundance of the four immune cell lines. We also compared with CIBERSORT absolute mode, which is a beta version in CIBERSORT website (S1 Fig). Since the CIBERSORT absolute mode is a beta version and leads to suboptimal results compared with CIBERSORT, we only focused on the comparisons with CIBERSORT. We show that FARDEEP gives the smallest SSE and the largest *R*^2^, which indicates the most accurate result (Fig 3).

### Synthetic dataset with added unknown content

**Fig 4:**
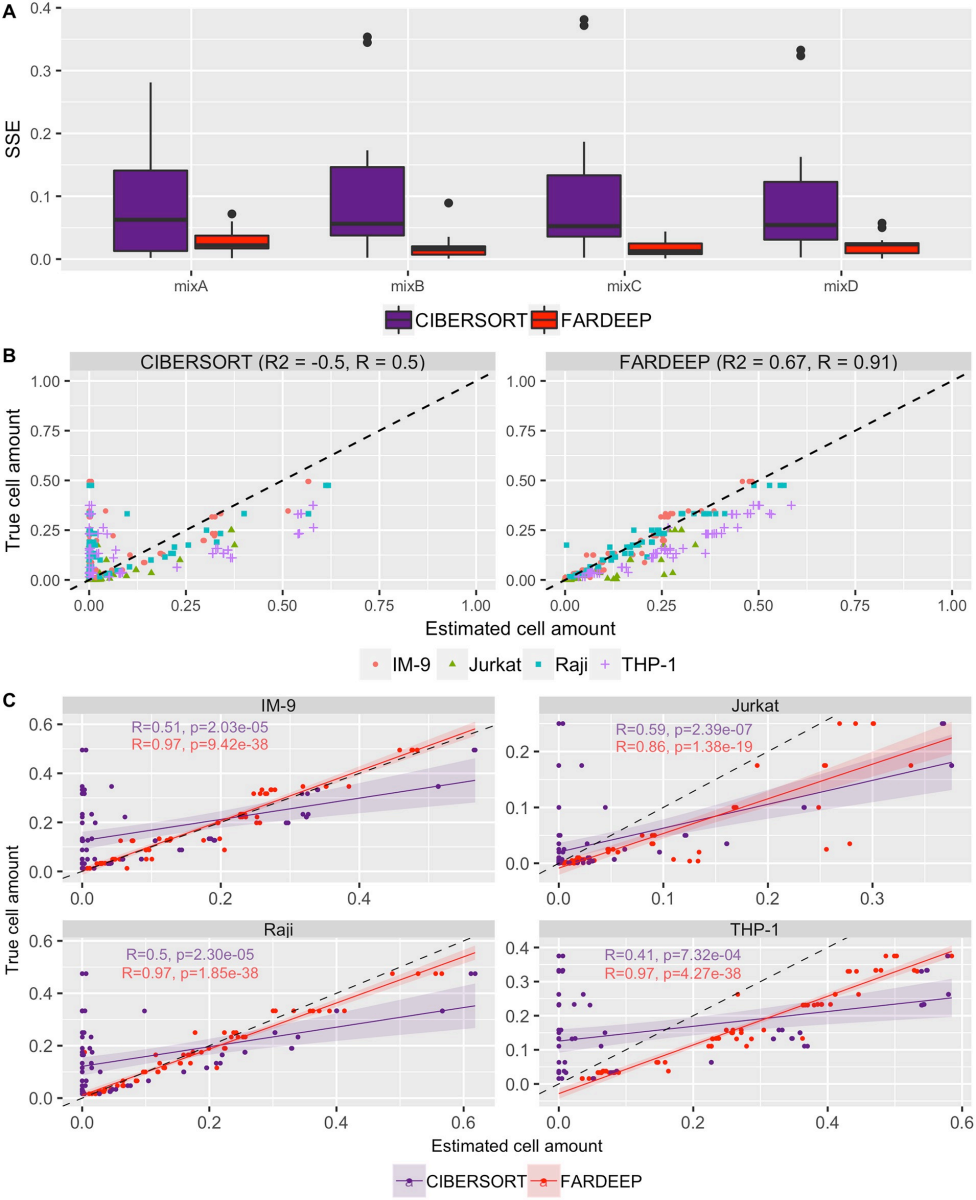
Performance comparison between CIBERSORT and FARDEEP on mixtures with unknown content. (A) SSE for various noise and abundance of tumor contents on 4 different mixtures. (B) Abundance of cell lines estimated by CIBERSORT and FARDEEP vs. Abundance of cell lines truly mixed. The *R*^2^ and *R* values are also reported in the top of the figures. (C) Deconvolution of individual cell subsets by FARDEEP and CIBERSORT. The correlation coefficients *R*, the corresponding *p*-values against the null hypothesis of *R* = 0, trend lines with 95% confidence intervals are shown in the figures. The black dashed line represents the perfect relationship between the estimate and the true cell abundances with slope 1 and intercept 0.

Both CIBERSORT and FARDEEP are robust deconvolution methods and show advantages in the above datasets, we next sought to compare their performances on mixtures with unknown content. We followed the simulation setting proposed by Newman *et al*. [12] and downloaded the signature gene file from CIBERSORT website. The mixture file was constructed from the four immune cell lines data, as mentioned in the previous section, and a colon cancer cell line HCT116 (average of GSM269529 and GSM269530 in GSE10650). Cancer cells were mingled into immune cells at different ratios {0%, 30%, 60%, 90%}. Noise was added by sampling from the distribution $2^{N(0,{\left(f\;{\text{log}}_{2}\left(s\right)\right)}^{2})}$, in which *f* ∈ {0%, 30%, 60%, 90%} and *s* is the standard deviation of original mixtures. By applying FARDEEP and CIBERSORT (without converting to percentage) on 64 mixtures, we found that FARDEEP remains an accurate estimation, while the tumor contents skew the results of CIBERSORT with larger deviation from the ground truth (Fig 4).

### Deconvolution performance on immune-cell-rich datasets

To evaluate the performance of FARDEEP in immune-cell-rich settings that are less affected by outliers, we downloaded and analyzed two gene expression datasets (GSE65135 [12] and GSE20300 [36]) generated from the Affymetrix Microarray, which is the same platform used to generate the signature matrix LM22. The GSE65135 dataset consists of (i) surgical lymph node biopsies of 14 follicular lymphoma patients and (ii) purified B and T cells from the tonsils of 5 healthy controls, and the GSE20300 dataset includes 24 blood samples from pediatric renal transplant patients. Flow cytometry or coulter counter data in these studies, which are presented in relative scales, are treated as ground truth. Thus, we normalized the estimated parameters of each method to the sum of 1 before comparison.

**Fig 5:**
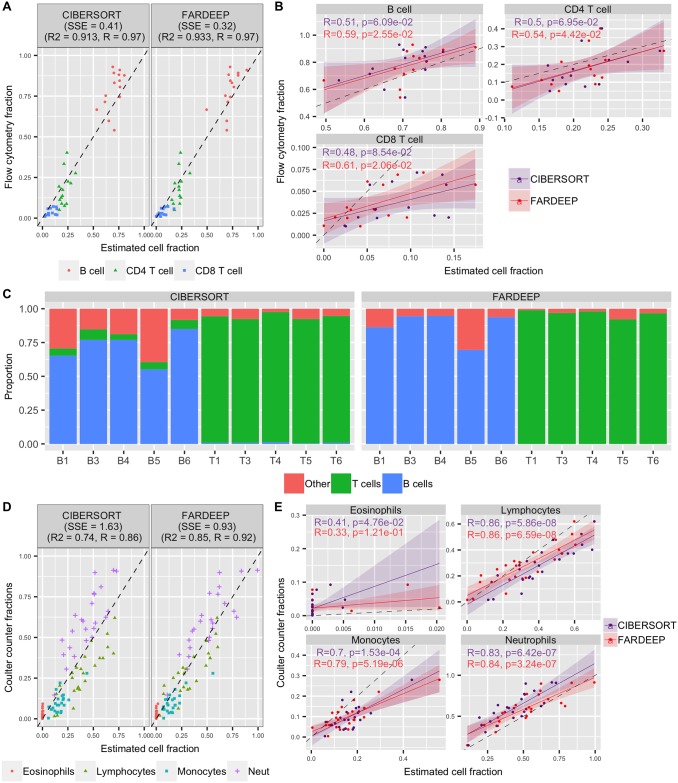
Performance assessment on Microarray data with SSE, *R*^2^ and *R*. The follicular lymphoma dataset is evaluated for (A) Overall performance and (B) individual cell subsets. (C) Normal tonsil dataset with purified B cells and T cells. The blood samples from pediatric renal transplant patients are evaluated for (D) Overall performance and (E) individual cell subsets. The black dashed line represents the perfect relationship between the estimate and the true cell abundances with slope 1 and intercept 0.

As shown in Fig 5A and 5B for case (i) of GSE65135 and Fig 5D and 5E for GSE20300, FARDEEP outperformed CIBERSORT in terms of *R*^2^, *R* and SSE, which is consistent with our findings with simulated datasets. For case (ii) of GSE65136, we estimated the immune cell composition for purified B and T cells with purity level exceeding 95% and 98%, respectively. For purified B cells, CIBERSORT tends to return non-zero estimates for T cell and a large proportion of other cell types, while FARDEEP gave almost all zero estimates for T cell and on average reduced the estimation error by 61%. Similarly, for the purified T cell, although CIBERSORT had a better performance compared to purified B cell, FARDEEP still significantly improves the estimation accuracy by reducing on average 48% of the estimation error (Fig 5C). Furthermore, as shown in S4 Table, FARDEEP detected gene *CD79A* and *BCL2A1* as outliers for most samples in case (i) of GSE65135. These two genes are known to have high expression levels in follicular lymphoma (B-cell lymphoma) cells [37].

Overall, even in specimens that are rich in immune cells without contamination by non-hematopoietic malignancy, FARDEEP still outperforms CIBERSORT in immune cell deconvolution.

### Deconvolution performance on RNA-seq datasets

**Fig 6:**
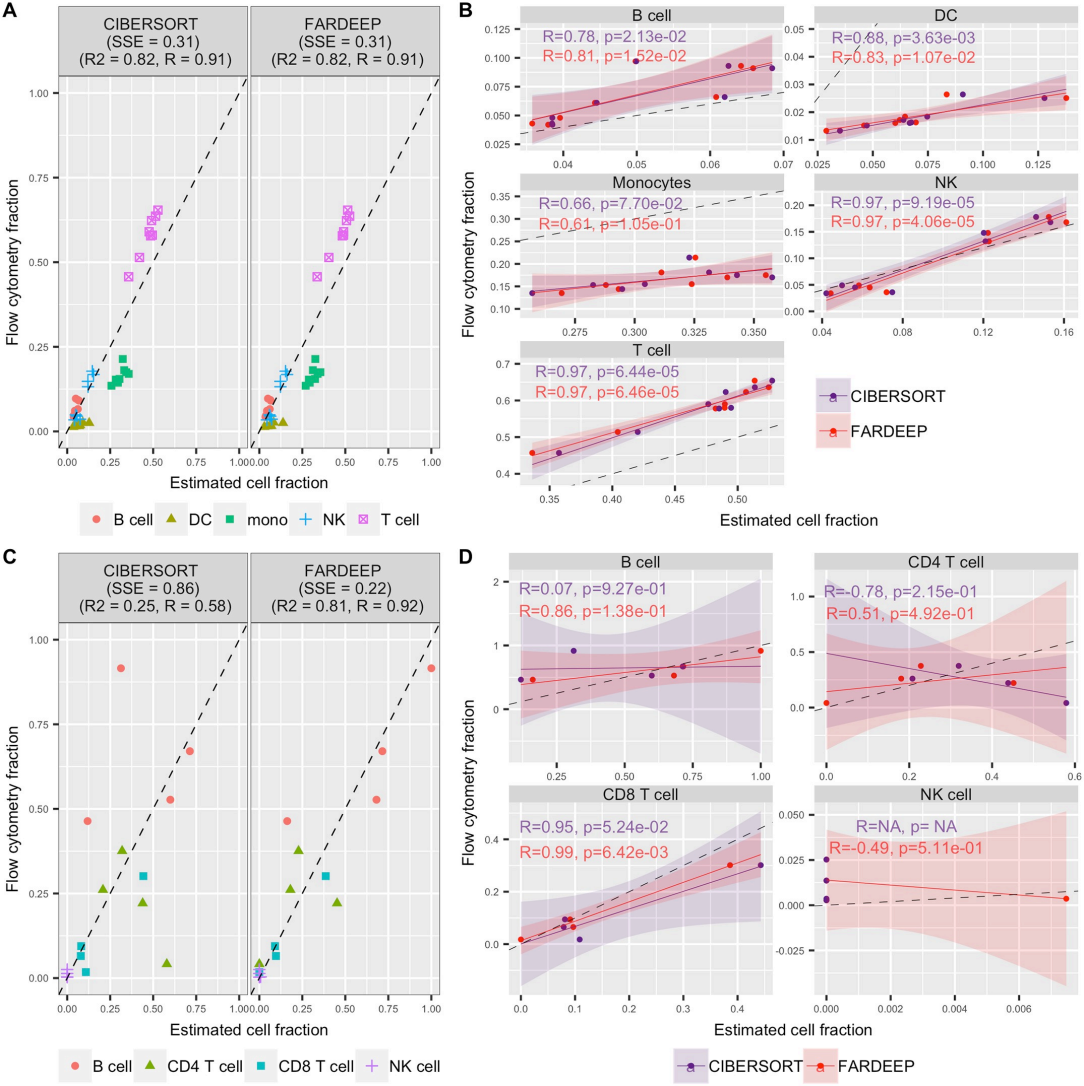
Gene-expression deconvolution performance of FARDEEP and CIBERSORT on RNA-seq data. (A) Overall and (B) individual cell subsets results for 8 PBMC samples collected from two vaccinated donors at different time points. (C) Overall and (D) individual cell subsets result for lymph node bulk samples. Correlation coeffient *R* and the correspoinding *p*-value are missing for CIBERSORT on NK cell because the CIBERSORT estimations are all zero. The black dashed line represents the perfect relationship between the estimate and the true cell abundances with slope 1 and intercept 0.

In addition to effectively handling Microarray data, FARDEEP can also deconvolve TILs using RNA-seq data when we replace the signature matrix LM22 with quanTIseq, a signature matrix generated from RNA-seq data containing ten different immune cell types [14]. We applied CIBERSORT and FARDEEP using signature matrix quanTIseq to peripheral blood mononuclear cell (PBMC) mixtures (GSE64655) generated by Hoek *et al*. [38], and lymph node bulk samples of 4 melanoma patients from GSE93722 [39]. Flow cytometry data in these studies are on a relative scale and are treated as ground truth. We normalized the estimated parameters of each method to a relative scale using (0.7) before comparison. The RNA-seq data are usually less noisy compared to Microarray, and PBMC datasets are usually clean with less unknown contents. Therefore, we expect FARDEEP and CIBERSORT will return comparable results, which is the case in Fig 6A and 6B. However, when dealing with noisier data containing more outliers such as lymph node bulk samples, FARDEEP obtained larger advantage over CIBERSORT as shown in Fig 6C and 6D.

### Ovarian serous cystadenocarcinoma and lung squamous cell carcinoma datasets

**Fig 7:**
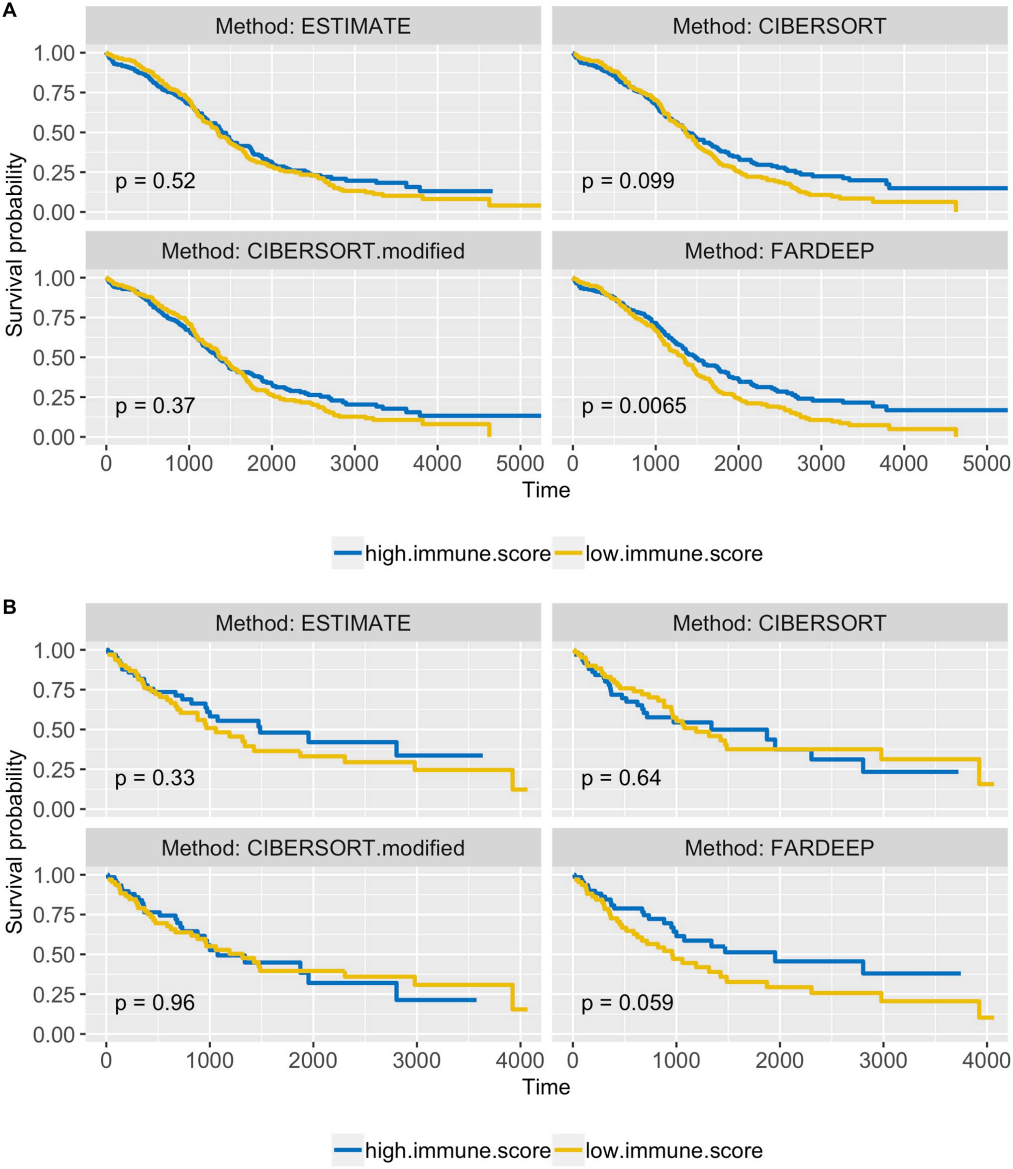
Kaplan-Meier survival curves are plotted based on ESTIMATE, FARDEEP- and CIBERSORT- assisted TIL profiling. Log-Rank test was applied to data classified into two groups according to whether immunoscore (ESTIMATE) or collective anti-tumor immune subsets (CIBERSORT and FARDEEP) was above or below the median. (A) 514 patients with ovarian cancer. (B) 133 patients with lung squamous cell carcinoma.

TME of solid carcinomas are different from a lymph node biopsy or peripheral blood, and the highly variable gene expression in cancer cells challenges the accuracy of immune cell deconvolution. It is well-established that immune infiltration profile serves as a promising prognosticator [4, 5]. Hence, we next utilized survival and gene expression data of ovarian cancer (OV) and lung squamous cell carcinoma (LUSC) from The Cancer Genome Atlas (TCGA) database to assess the prognostic relevance of different deconvolution methods. These two datasets were chosen because only LM22 not the RNA-seq based signature matrix quanTIseq includes *γδ* T cells, and OV and LUSC from TCGA datasets are the only two cancer types with Affymetrix microarray data. Using gene expression data (n = 514 for OV and n = 133 for LUSC), we estimated the immunoscore using ESTIMATE proposed by yoshihara *et al*. [40], TILs proportion using CIBERSORT, as well as TILs subset scores using CIBERSORT (without converting to percentage) and FARDEEP. Cold tumors typically harbor lower numbers of CD8^+^ T cells, *γδ* T cells, M1-like macrophages, and NK cells [11, 41–43]. Thus, we calculated an anti-tumor immune subsets score by the summation of CD8^+^ T cells, *γδ* T cells, M1-macrophages, and NK cells. Then, we partitioned the patients into two groups with equal size using the median of either the immunoscore (ESTIMATE) or anti-tumor immune subsets score (CIBERSORT and FARDEEP). We compared the survival curves between the two groups. As shown in Fig 7, FARDEEP most effectively separates patients into high- and low- risk groups with the smallest p-value (p-value = 0.0065 and 0.059 for OV and LUSC respectively). Recently, CIBERSORT website supports a beta-version of an absolute mode for cell deconvolution. We also included CIBERSORT absolute mode in this survival analysis and showed that it returned a better result (p-value = 0.037) compared to the relative mode in the OV dataset. FARDEEP shows a stronger performance with a smaller p-value under this setting (S2 Fig). These results demonstrated that the TIL subset scores could provide additional clinical-relevant information compared to the relative abundance.

In addition, we expected the summation of these TIL subset scores would negatively correlate with tumor purity. To prove this hypothesis, we calculated the summation of 22 TIL subset scores for both OV and LUSC datasets and correlated them with the tumor purity estimated from consensus measurement of purity estimations (CPE) [44]. Even without taking account of stromal cells, as shown in S3 Fig. the summation of TIL subset scores is negatively correlated with tumor purity.

Next, we sought to investigate whether outlier removal reduces contamination by transcripts from cancer cells. We first identified those top outlier-genes, which were consistently removed by FARDEEP in the OV dataset and obtained the average expression values of those outlier-genes from OV cell lines in GSE32474 [45]. As shown in S3 Table, most of these outlier-genes have high expression in cancer cell lines. For example, *CXCL10* gene encodes an important chemokine to recruit CD8^+^ T cells and is also highly expressed in ovarian cancer cells. Thus, although some genes in LM22 may play a role in immune cells, they may be also highly expressed and variable among cancer cells. Such cross-contamination likely skews immune deconvolution analysis. As shown in S3 Table, FARDEEP successfully detected and removed those genes, leading to a more robust and accurate deconvolution analysis.

## Discussion

The cancer immune microenvironment has emerged as a critical prognostic dimension that modulates patient responses to neoadjuvant therapy. However, the current clinical TNM staging system does not have a consistent method to stratify cancers based on their immunogenicity. The recent study shows that the RNA-seq datasets of whole tumors contain valuable prognostic information to assess the cancer-immunity interactions [12, 46]. But the current methods to extract immune signatures are susceptible to the frequent outliers in the datasets, leading to less effective identification of cold tumors. Based on support vector regression, CIBERSORT is one of the most popular robust deconvolution methods. However, this model does not include an intercept to capture possible contribution from other cell types and performs a z-normalization to the data before fitting the regression model, which introduces biases into the output. Discussion of the effect of Z-score normalization for CIBERSORT is included in S1 Text. In this study, we developed a new machine learning tool, FARDEEP, to streamline the removal of outliers and increase the robustness of gene-expression profile deconvolution. Robustness is an indispensable feature to solve a problem of deconvolution because gene expression data are frequently contaminated by a large amount of outliers. FARDEEP solves the deconvolution problem in a robust way because this tool evaluates all outliers across the datasets and then examines the true immune gene signature using non-negative regression. This feature is especially useful to analyze tumors with significant non-hematopoietic tumor components. Interestingly, although FARDEEP and the current robust methods can both tackle immune-cell-rich specimens such as lymph node and PBMCs, FARDEEP exhibits improved prognostic potential when dealing more complex datasets with significant carcinoma cell content.

Although FARDEEP provides a robust computational algorithm to better solve the gene-expression deconvolution problem with noisy datasets, its performance and application rely on the choice of the signature matrix. To avoid estimation bias, it is important to choose the signature matrix derived from the same platform as the mixture matrix. For example, if dealing with gene expression data measured by Affymetrix HGU133A, we should use LM22, but if dealing with RNA-seq data, the signature matrix quanTIseq is preferred. Overall, here we show that FARDEEP is a powerful and rapid machine learning tool that outperforms existing robust methods for gene deconvolution in datasets with significant heavy-tailed noise. FARDEEP provides a new technology to interrogate cancer immunogenomics and more accurately map the immune landscape of solid tumors.

## Supporting information

S1 Table Parameters of FARDEEP. To show that FARDEEP is not sensitive for different values of *α*_1_, and *α*_2_ with tuned value of *k*, we simulated a dataset with sample size *n* = 500, number of predictors *p* = 20, normal distributed error and 20% outliers using the same setting of *in silico* simulation in the paper. Then we ran FARDEEP with following setting and get the number of detected outliers, true and false positive rate: (1) Take *α*_2_ = 1.1, change *α*_1_ from 0.1 to 0.5 by 0.05 and tune *k* using BIC*. (2) Take *α*_1_ = 0.1, change *α*_2_ from 1.1 to 2 by 0.1 and tune *k* using BIC*. (3) Take *α*_1_ = 0.1, *α*_2_ = 1.5, and change *k* from 1 to 10 by 0.1. We can see that the accuracy of the result stays stable with a well tuned *k*.
(PDF)

S2 Table Performance of FARDEEP, CIBERSORT, NNLS, PERT and DCQ methods under different simulation settings with outliers. (PDF)

S3 Table The outlier genes detected by FARDEEP and their average expression values in 7 ovarian cancer cell lines for TCGA OV dataset. Here we listed all genes with removal frequency larger than 50% among 514 samples.
(PDF)

S4 Table Table of removed outlier genes for each real datasets. (XLSX)

S1 Fig Applying different deconvolution approaches on the gene expression data of IM-9, Jurkat, Raji, THP-1 and the mixture of these four immune cell lines with known proportion (MixA, MixB, MixC, MixD). All of the mixtures were performed and measured in triplicate. (A) SSE of coefficients for FARDEEP, CIBERSORT, CIBERSORT under absolute mode (CIBERSORT.abs), NNLS, PERT, DCQ. (B) Abundance of cell lines estimated from different deconvolution approaches vs. Abundance of cell lines truly mixed. The *R*^2^ and *R* values are also reported at the top of the figures. The black dashed line represents the perfect relationship between the estimate and the true cell abundances with slope 1 and intercept 0.
(EPS)

S2 Fig Kaplan-Meier survival curves are plotted based on CIBERSORT (absolute mode)- assisted TIL profiling. Patients were classified into two groups according to whether immunoscore (ESTIMATE) or collective anti-tumor immune subsets was above or below the median. Log-Rank test was applied to obtain the p-value. (A) 514 patients with ovarian cancer. (B) 133 patients with lung squamous cell carcinoma.
(EPS)

S3 Fig FARDEEP score of TCGA OV and LUSC datasets are calculated from the summation of 22 TIL subset scores, which show highly negative correlations to the consensus measurement of purity estimations (CPE). (EPS)

S1 Text Discussion for the effect of Z-score normalization. (PDF)

S2 Text Discussion for the importance of TIL subset scores. (PDF)

S3 Text Discussion for the *X* related outliers and correlated responses. (PDF)
