# Supplementary material for: Fast and robust deconvolution of tumor infiltrating lymphocyte from expression profiles using least trimmed squares
Source: PLoS Comput Biol. 2019 May 6;15(5):e1006976. doi: 10.1371/journal.pcbi.1006976 (PMC6522071; doi:10.1371/journal.pcbi.1006976)
Supplement: S2 Table — (PDF) [file pcbi.1006976.s002.pdf]

Proportion of outliers	Mean of SSE (Standard deviation of SSE)				
	FARDEEP	CIBERSORT	NNLS	PERT	DCQ
2%	1.68e-7 (1.21e-7)	5.07 (1.84)	2.70 (7.01)	6.37 (1.50)	3.58 (1.64)
4%	2.20e-7 (1.09e-7)	4.76 (1.42)	0.35 (0.67)	6.32 (1.47)	3.17 (0.54)
6%	2.35e-7 (2.17e-7)	5.32 (1.84)	1.80e1 (5.35e1)	6.45 (1.63)	9.00 (1.83e1)
8%	2.31e-7 (1.22e-7)	5.58 (1.52)	6.03 (1.16)	6.42 (1.47)	3.85 (2.17)
10%	2.18e-7 (2.19e-7)	5.08 (1.25)	2.09 (2.35)	6.34 (1.45)	2.98 (0.58)
12%	1.51e-7 (4.86e-8)	5.95 (2.11)	3.33e1 (6.29e1)	6.42 (1.54)	5.45 (5.24)
14%	2.38e-6 (1.01e-7)	5.87 (1.08)	9.62 (2.25e1)	6.42 (1.48)	3.71 (2.77)
16%	3.70e-7 (3.00e-7)	6.42 (1.44)	8.76 (1.06e1)	6.55 (1.26)	4.33 (4.35)
18%	2.83e-7 (9.38e-8)	6.51 (1.33)	1.64e2 (3.56e2)	6.59 (1.61)	2.02e1 (3.68e1)
20%	4.93e-7 (4.08e-7)	5.46 (1.44)	2.72 (4.37)	6.39 (1.41)	2.98 (0.78)
22%	5.99e-7 (3.51e-7)	6.37 (1.92)	1.98e1 (2.43e1)	6.43 (1.44)	5.13 (4.18)
24%	4.00e-6 (7.16e-6)	6.00 (1.81)	7.36e1 (2.00e2)	6.46 (1.46)	8.95 (1.66e1)
26%	1.02e-6 (1.23e-6)	6.36 (1.11)	6.25 (5.17)	6.46 (1.41)	3.33 (1.92)
28%	8.14e-7 (9.14e-7)	6.61 (1.74)	2.37e1 (3.55e1)	6.60 (1.46)	6.51 (6.30)
30%	1.54e-6 (2.04e-6)	6.59 (1.13)	1.94e1 (2.26e1)	6.48 (1.34)	6.58 (6.51)
32%	5.12e-6 (7.68e-6)	6.82 (1.51)	6.66e1 (1.08e2)	6.62 (1.51)	2.17e1 (3.81e1)
34%	3.03e-6 (3.56e-6)	6.61 (1.66)	2.34e1 (3.88e1)	6.43 (1.38)	6.42 (8.53)
36%	3.36e-5 (6.32e-5)	6.60 (1.67)	4.36e1 (4.24e1)	6.47 (1.46)	9.90 (9.98)
38%	4.53e-6 (5.91e-6)	6.66 (1.54)	1.29e1 (1.60e1)	6.41 (1.44)	3.65 (2.18)
40%	1.01e-5 (1.19e-5)	6.69 (1.87)	7.34e1 (1.58e2)	6.53 (1.55)	2.37e1 (5.54e1)
42%	1.06e-4 (2.96e-4)	6.90 (1.53)	1.18e1 (1.00e1)	6.56 (1.44)	3.37 (1.58)
44%	3.04e-5 (4.10e-5)	6.90 (1.43)	3.96e1 (5.08e1)	6.55 (1.38)	1.11e1 (1.30e1)
46%	6.44e-6 (7.10e-6)	6.60 (1.28)	1.98e1 (2.99e1)	6.41 (1.42)	7.03 (9.26)
48%	1.47e-4 (1.78e-4)	7.10 (1.68)	8.03e1 (1.55e2)	6.74 (1.61)	2.14e1 (3.81e1)
50%	5.28e-5 (5.26e-5)	6.86 (1.58)	2.97e1 (3.01e1)	6.57 (1.35)	6.88 (7.83)
In all	Range of R^2 (lower bound $\sim$ upper bound)				
	FARDEEP	CIBERSORT	NNLS	PERT	DCQ
	1.00 $\sim$ 1.00	-3.87 $\sim$ -0.47	-7.10e2 $\sim$ 1.00	-3.86 $\sim$ -1.13	-9.88e1 $\sim$ 0.45
	Range of R (lower bound $\sim$ upper bound)				
	FARDEEP	CIBERSORT	NNLS	PERT	DCQ
	1.00 $\sim$ 1.00	0.58 $\sim$ 1.00	-0.38 $\sim$ 1.00	-0.38 $\sim$ 0.76	-0.36 $\sim$ 0.86
