# Supplementary material for: Fast and robust deconvolution of tumor infiltrating lymphocyte from expression profiles using least trimmed squares
Source: PLoS Comput Biol. 2019 May 6;15(5):e1006976. doi: 10.1371/journal.pcbi.1006976 (PMC6522071; doi:10.1371/journal.pcbi.1006976)
Supplement: S3 Table — Here we listed all genes with removal frequency larger than 50% among 514 samples. (PDF) [file pcbi.1006976.s003.pdf]

Gene	Frequency	NCI-ADR-RES	OVCAR-3	OVCAR-4	OVCAR-5	OVCAR-8	SK-OV-3	IGROV1
CHI3L1	96%	9.76	37.4	1149.51	4.32	4.28	7.78	28.91
APOBEC3G	84%	654	24.5	32.12	242.22	612.56	98.77	90.38
CXCL10	84%	25.05	66.55	15.25	26.47	46.24	47.85	45.19
IFI44L	79%	37.15	182.44	15.44	58.11	61.26	66.49	4.63
SLC12A8	79%	291.4	426.97	342.54	81.4	432.2	77.93	89.91
RGS1	75%	3.63	3.47	2.51	3.28	10.2	7.74	6.82
C1orf54	71%	80.14	33.86	117	19.46	98.08	46.11	98.27
FZD3	69%	65.76	394.81	216.9	74.34	108.69	477.65	878.79
PNOC	68%	31.96	4.63	28.14	25.08	16.63	40.56	314.87
STXBP6	66%	537.71	2231.15	464.33	217.93	240.62	4.99	6.41
TRIB2	66%	140.69	568.85	108.99	1910.09	113.24	439.38	216.98
GSTT1	63%	749.88	1787.77	19.09	994.12	604.82	518.46	1176.07
MS4A6A	61%	29.42	31.78	16.15	21.76	40.23	23.15	31.04
SPOCK2	59%	117.32	326.4	94.08	213.96	76.59	88.95	145.94
TCF7	57%	63.48	79.55	92.49	238.59	93.24	119.39	80.23
PPFIBP1	56%	391.73	1279.24	819.14	828.22	277.91	415.94	615.33
RSAD2	56%	17.54	46.41	33.83	86.84	36.56	30.13	18.02
PLEKHF1	54%	120.38	2345.08	522.76	167.33	284.82	687.72	322.99
FCGR2B	53%	7.64	30.34	30.8	8.08	14.7	15.7	32.79
HLA-DQA1	52%	15.65	27.75	32.08	6.74	29.95	19.1	36.81
LAMP3	52%	250.12	103.46	1963.43	165.71	1186.43	179.73	366.39
NCF2	51%	183.96	9.12	87.42	317.48	423.67	21.72	237.07
