# Supplementary material for: Fast and robust deconvolution of tumor infiltrating lymphocyte from expression profiles using least trimmed squares
Source: PLoS Comput Biol. 2019 May 6;15(5):e1006976. doi: 10.1371/journal.pcbi.1006976 (PMC6522071; doi:10.1371/journal.pcbi.1006976)
Supplement: S1 Text — (PDF) [file pcbi.1006976.s008.pdf]

Effect of Z-score normalization

CIBERSORT employed a Z-score normalization on both $\boldsymbol{y}$ (gene expression profile of tumor) and $\boldsymbol{X}$ (expression values of signature genes) to stabilize the Support Vector Regression algorithm. To assess the effect of Z-score normalization, we performed a brief mathematical derivation together with several simulations as follows:

$$\boldsymbol{y} = \boldsymbol{X}\boldsymbol{\beta} + \boldsymbol{\epsilon}, \tag{1}$$

where $\boldsymbol{\epsilon}$ is the noise. First, CIBERSORT performs Z-score on both y and X to get

$$\boldsymbol{y}^* = \frac{\boldsymbol{y} - \bar{y}\mathbf{1}}{s_y},$$
$$\boldsymbol{X}^* = \frac{\boldsymbol{X} - \bar{X}\mathbf{1}\mathbf{1}^T}{s_X},$$

where $\bar{y} = \sum_{i=1}^{n} y_i/n$; $\bar{X} = \sum_{i=1}^{n}\sum_{j=1}^{p} x_{ij}/(np)$; s_y and s_X are the sample standard deviation of $\{y_i\}_{i=1}^{n}$ and $\{x_{ij}\}$ respectively. Then, CIBERSORT utilizes Support Vector Regression (SVR) on $\boldsymbol{y}^*$ and $\boldsymbol{X}^*$ to obtain the coefficients. Note that in practice s_y and $\bar{y}$ differs from s_X and $\bar{X}$ respectively. Thus, the SVR coefficients based on $\boldsymbol{y}^*$ and $\boldsymbol{X}^*$ are much less likely to equal those based on $\boldsymbol{y}$ and $\boldsymbol{X}$.

To demonstrate the point, we simulated several mixtures based on the first 9 columns of LM22 signature matrix with $\beta \in \mathbb{R}^9 = (2, 1.5, 1.2, 1, 0.8, 0.6, 0.5, 0.3, 0.2)'$ and random noise $\epsilon \sim 2^{\mathcal{N}(0,(0.3\log_2(s))^2)}$, where s is the standard deviation of the original mixtures. As shown in Figure A1, the estimates from SVR with Z-score normalization showed skewness from the truths which are denoted as red dashed lines in the figures. Thus, Z-score normalization in CIBERSORT blocks its way to getting the absolute cell abundance. Also, although the SVR without Z-score normalization performed well in this simulation, it is very slow and may not converge for the high-dimensional signature matrix, and the variables with greater numeric ranges will dominate those with smaller numeric ranges as discussed in Chang and Lin (2011); Hsu *et al.* (2003). This is also the reason that we only include the first 9 columns of LM22 in this simulation.

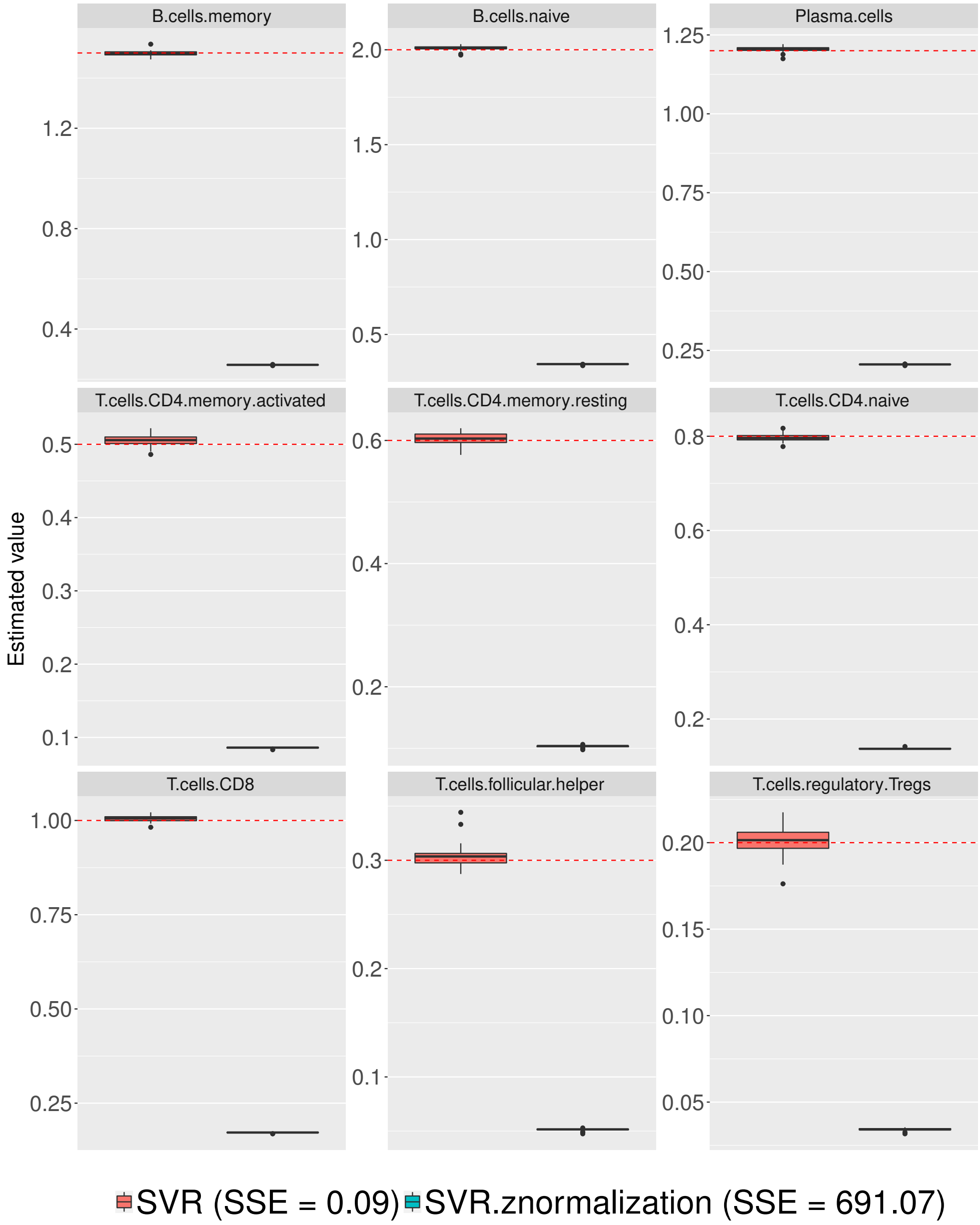

Figure A1: Effect of Z-score normalization. Red dashed line indicates the true absolute abundance of each cell. SVR is the common Support vector regression without normalization and SVR.znormalization is the method used in CIBERSORT.

References

Chang, C.-C. and Lin, C.-J. (2011). Libsvm: A library for support vector machines. *ACM Transactions on Intelligent Systems and Technology*, **2**, 27:1–27:27.

Hsu, C.-W., Chang, C.-C., and Lin, C.-J. (2003). A practical guide to support vector classification. *Technical report, Taipei*.
