# Supplementary material for: Fast and robust deconvolution of tumor infiltrating lymphocyte from expression profiles using least trimmed squares
Source: PLoS Comput Biol. 2019 May 6;15(5):e1006976. doi: 10.1371/journal.pcbi.1006976 (PMC6522071; doi:10.1371/journal.pcbi.1006976)
Supplement: S2 Text — (PDF) [file pcbi.1006976.s009.pdf]

Importance of TIL subset scores

The TIL subset score is a good indicator for the absolute abundance of TIL and is an important scale to evaluate the immune level for cancer patients. To demonstrate this, we simulate two mixtures without noise using the pure immune cell lines (Jurkat, IM-9, Raji and THP-1) expression profiles from GSE11103 and two different cancer cell lines (Colon cancer: HCT116, Melanoma: MDA-MB-435) expression profile from GSE10650 and GSE32474 respectively as below,

$$\text{MIX1} = 0.2\text{cancer cell line} + 0.2\text{Jurkat} + 0.2\text{IM-9} + 0.2\text{Raji} + 0.2\text{THP-1}$$
$$\text{MIX2} = 0.8\text{cancer cell line} + 0.05\text{Jurkat} + 0.05\text{IM-9} + 0.05\text{Raji} + 0.05\text{THP-1}$$

where cancer cell line can be either HCT116 or MDA-MB-435. Then we applied CIBERSORT and FARDEEP with the signature matrix generated by Jurkat, IM-9, Raji, and THP-1 which could be downloaded from CIBERSORT website. The outputs are shown below in Table A1. CIBERSORT gives only the relative proportion as around 0.25 for both mixtures even if mixture 2 has less immune contents than the mixture 1. In contrast, FARDEEP shows its ability to accurately estimate the exact abundance of immune cells through TIL subset scores because they are the direct estimate from the linear model. Also, we can derive relative abundance (fraction) among immune cells from TIL subset scores by

$$\tilde{\beta}_j = \frac{\hat{\beta}_j}{\sum_{k=1}^{p} \hat{\beta}_j},$$

where $\hat{\beta}_j$ is the jth TIL subset score. As shown in Table A1, the relative abundance results derived from TIL subset scores still outperform CIBERSORT because

a. although most of the probesets in the signature matrix have high expression in the immune cell lines and low expression in the cancer cell line (HCT116 and MDA-MB-435), several signature probesets are highly expressed in HCT116 or MDA-MB-435 (Figure A1) which likely introduce an estimation bias. FARDEEP automatically removes those outlier probesets before estimating the abundance of the immune cells, as shown in Figure A1, hence delivering a better estimate;

b. compared to CIBERSORT, FARDEEP includes an extra intercept in the regression model, which could capture the background effect from non-immune content, such as tumor cells.

Interestingly, even with different ratios of cancer cells in MIX1 and MIX2 regardless of the cancer types, FARDEEP detected the same set of outliers from these two mixtures, but there are 137 and 151 outliers from HCT116 mixture and MDA-MB-435 mixture respectively from which 90 outliers are shared. This result suggests that different cancer cell line has distinct high expressed genes, and the outlier-genes strongly depend on the sample type.

Method	Sample	HCT116				MDA-MB-435			
		Jurkar	IM-9	Raji	THP-1	Jurkar	IM-9	Raji	THP-1
Truth	MIX1	0.2	0.2	0.2	0.2	0.2	0.2	0.2	0.2
	MIX2	0.05	0.05	0.05	0.05	0.05	0.05	0.05	0.05
CIBERSORT	MIX1	0.2512	0.2493	0.2493	0.2503	0.2507	0.2514	0.2480	0.2499
	MIX2	0.2682	0.2404	0.2387	0.2527	0.2801	0.2649	0.2153	0.2398
FARDEEP	MIX1	0.2004	0.1995	0.1999	0.1999	0.2013	0.2002	0.2000	0.2002
	MIX2	0.0515	0.0479	0.0498	0.0497	0.0552	0.0509	0.0498	0.0510
FARDEEP (relative prop)	MIX1	0.2505	0.2494	0.2500	0.2500	0.2511	0.2497	0.2494	0.2498
	MIX2	0.2588	0.2410	0.2502	0.2500	0.2667	0.2459	0.2409	0.2465

Table A1: This table shows the results from CIBERSORT and FARDEEP on two mixtures (MIX1 and MIX2) with HCT116 and MDA-MB-435 respectively. FARDEEP (relative prop) was gotten by normalize the default outputs to sum to 1.

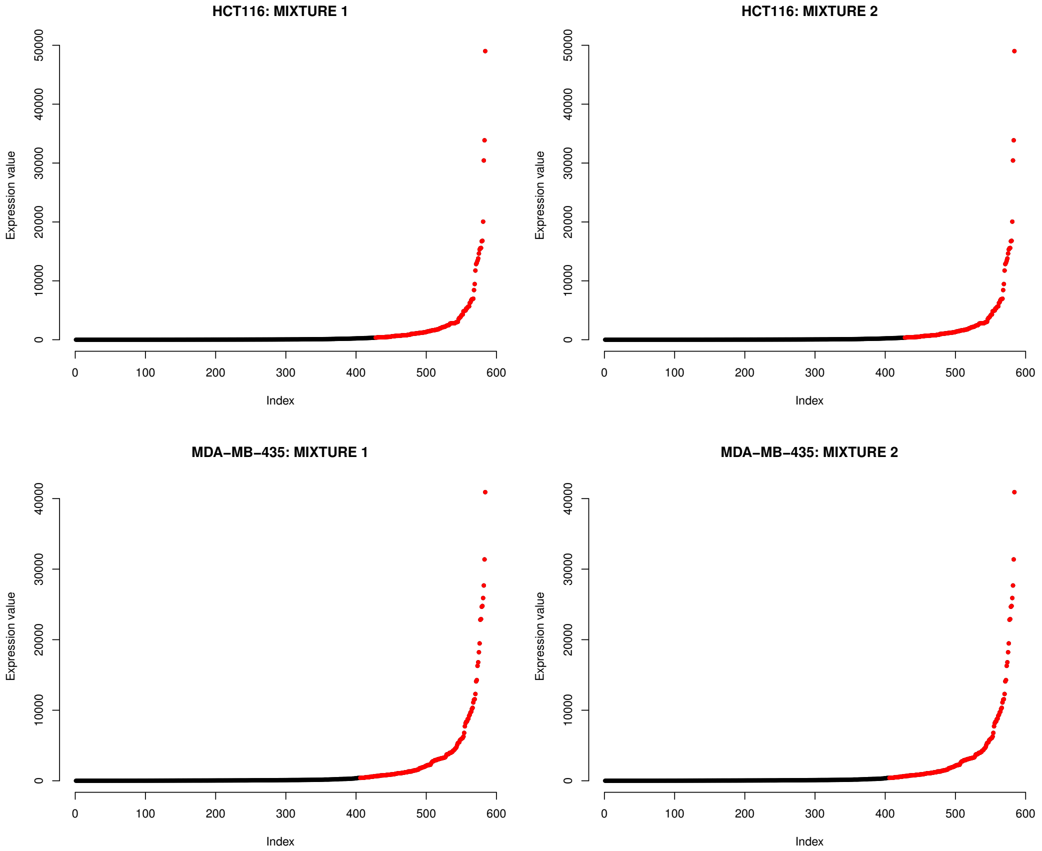

Figure A1: The y-axis are the sorted expression value of HCT116 and MDA-MB-435 cell line for 584 probesets. The probes which have been detected as outlier by FARDEEP are marked with color red.
