# Supplementary material for: Fast and robust deconvolution of tumor infiltrating lymphocyte from expression profiles using least trimmed squares
Source: PLoS Comput Biol. 2019 May 6;15(5):e1006976. doi: 10.1371/journal.pcbi.1006976 (PMC6522071; doi:10.1371/journal.pcbi.1006976)
Supplement: S3 Text — (PDF) [file pcbi.1006976.s010.pdf]

1 $\boldsymbol{X}$ related outliers

To evaluate the performance of FARDEEP under a more complex outlier construction, we simulated datasets 50 times follow the setting in section of "*in silico* simulation with varied error types", but let the outliers follow a non-central t-distribution with 1 degree of freedom and a non-centrality parameter being $10 \cdot \max(\boldsymbol{x_i})$ for the ith gene, where $\boldsymbol{x_i}$ is the ith row of $\boldsymbol{X}$. In this way, amplitude of the outliers for the ith gene will depend on $\boldsymbol{x_i}$. The results Figure A1, Figure A2 and Table A1 show that FARDEEP dominates the performances of other methods and keeps good accuracy of outlier detection.

Table A1: Tuned k for FARDEEP with the adjusted BIC. We simulated normally distributed errors and heavy-tailed errors respectively for different proportion of outliers and computed true positive rate and false positive rate to evaluate the tunning result.

Percentage of outliers		5%	10%	20%	30%
Normal	True positive rate	1	1	1	1
	False positive rate	0.007	0.02	0.05	0.06
	Parameter (mean of k)	2.53	1.32	1.16	1.09
Heavy-tailed	True positive rate	1	1	1	1
	False positive rate	0.06	0.06	0.13	0.17
	Parameter (mean of k)	2.05	1.24	1.10	1.04

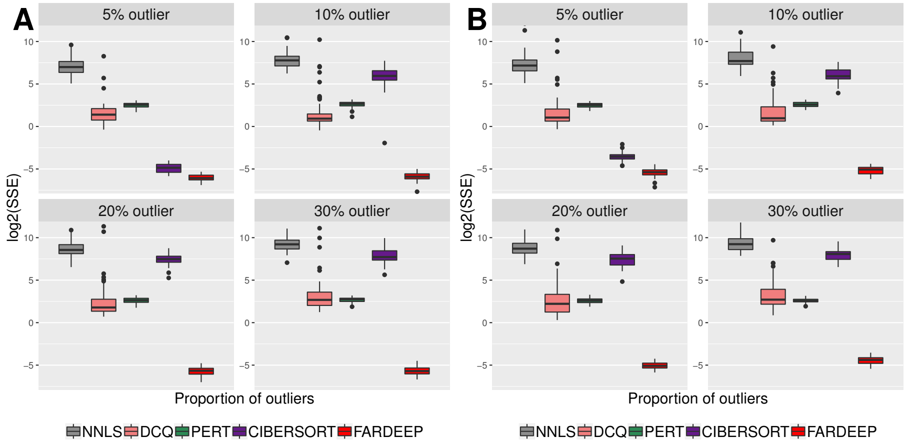

Figure A1: SSE of coefficients for different approaches based on simulations with $\boldsymbol{X}$ related outliers. We simulated different percentage of outliers ($\{5\%, 10\%, 20\%, 30\%\}$) and compared the SSE for coefficients applying NNLS, DCQ, PERT, CIBERSORT, and FARDEEP. (A) random error with standard normal distribution, (B) random error with t-distribution.

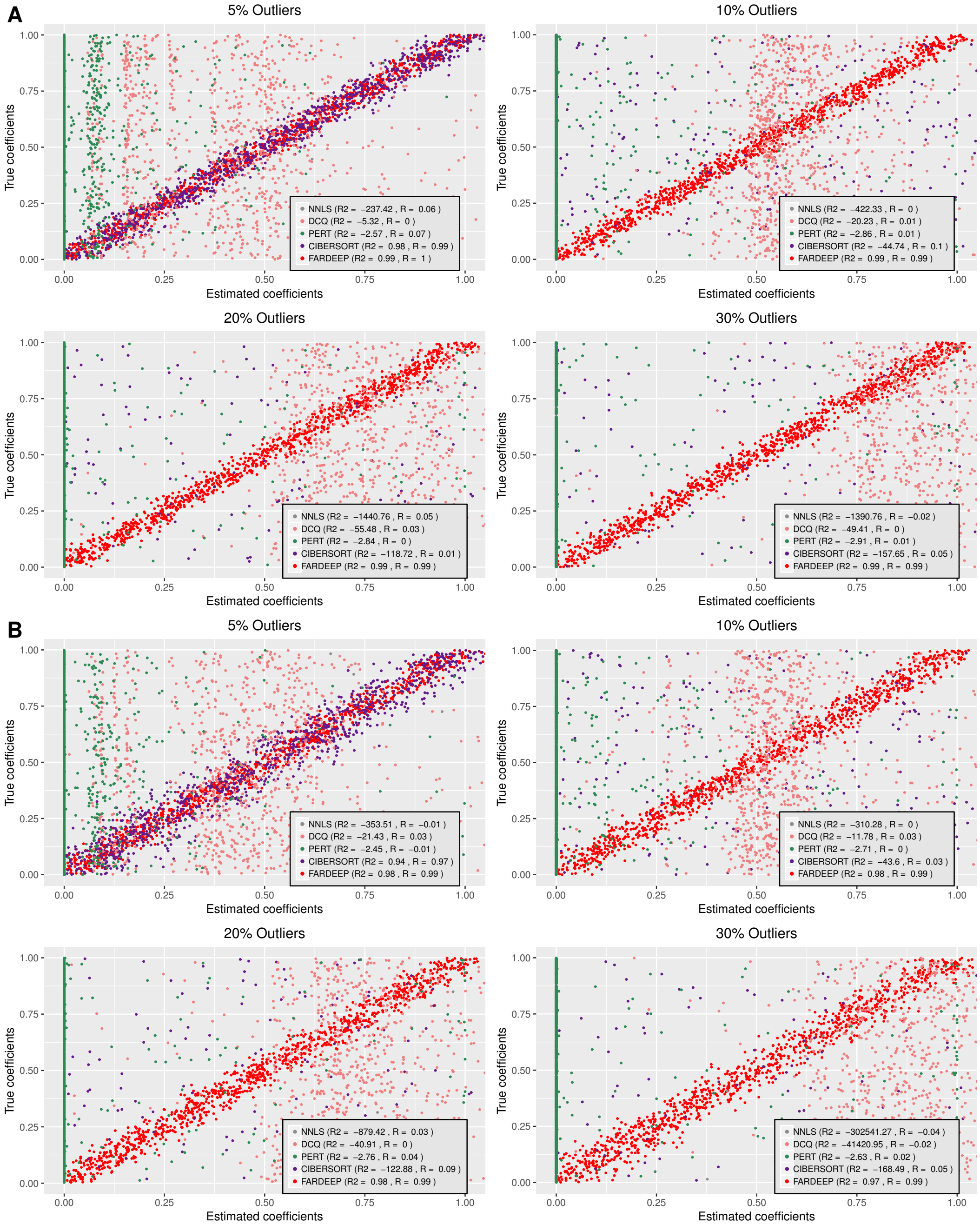

Figure A2: Compare the estimation accuracy of different deconvolution approaches for simulations with $\boldsymbol{X}$ related outliers (the values in parentheses are R^2 and R). Based on $\{5\%, 10\%, 20\%, 30\%\}$ percentage of outliers, we computed R^2 to evaluate how well are the estimators fit for a straight line $\hat{\boldsymbol{\beta}} = \boldsymbol{\beta}$. (A) random error with standard normal distribution, (B) random error with t-distribution.

2 Correlated genes

Gene-expression deconvolution problem can be expressed as a linear regression model (**??**). Sometimes the expression values among different genes are highly correlated. To evaluate the performance of FARDEEP under the violation of independent errors. We have simulated datasets 50 times with different proportion of outliers exactly following the simulation setting in section "*in silico* simulation with varied error types" of the paper except that the error term

$$\boldsymbol{\epsilon} \sim \mathcal{N}(\mathbf{0}, \boldsymbol{\Sigma}), \quad \text{where } \Sigma_{ij} = \begin{cases} 1 & i = j, \\ 0.7 & 1 \leq i, j \leq 20 \text{ and } i \neq j, \\ 0.5 & 21 \leq i, j \leq 40 \text{ and } i \neq j, \\ 0 & \text{others.} \end{cases}$$

Figure A3, A4 and Table A2 show that FARDEEP outperforms other approaches and keeps its accuracy under the correlated random errors.

Percentage of outliers	5%	10%	20%	30%
True positive rate	1	1	1	1
False positive rate	0.009	0.01	0.02	0.06
Parameter (mean of k)	3.53	2.48	1.45	1.19

Table A2: We simulated correlated errors with different proportion of outliers for FARDEEP, and computed true positive rate and false positive rate to evaluate the tunning result.

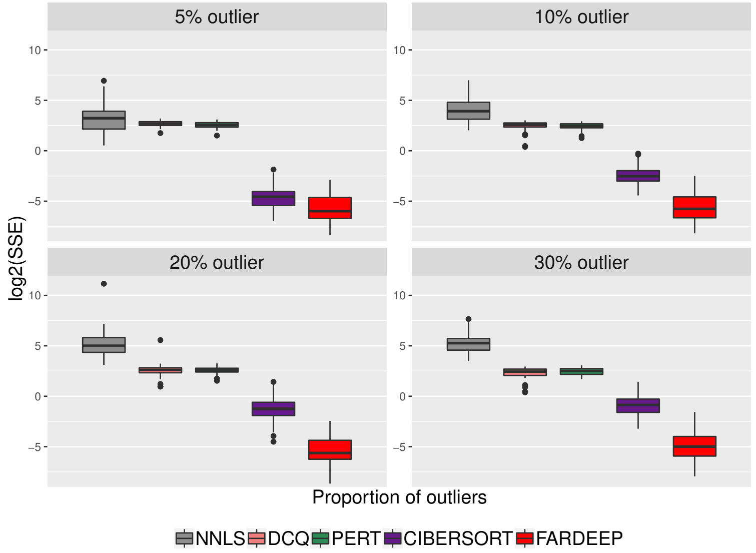

Figure A3: SSE of coefficients for different approaches based on simulations with correlated random errors. We simulated different percentage of outliers ($\{5\%, 10\%, 20\%, 30\%\}$) and compared the SSE for coefficients applying NNLS, DCQ, PERT, CIBERSORT, and FARDEEP.

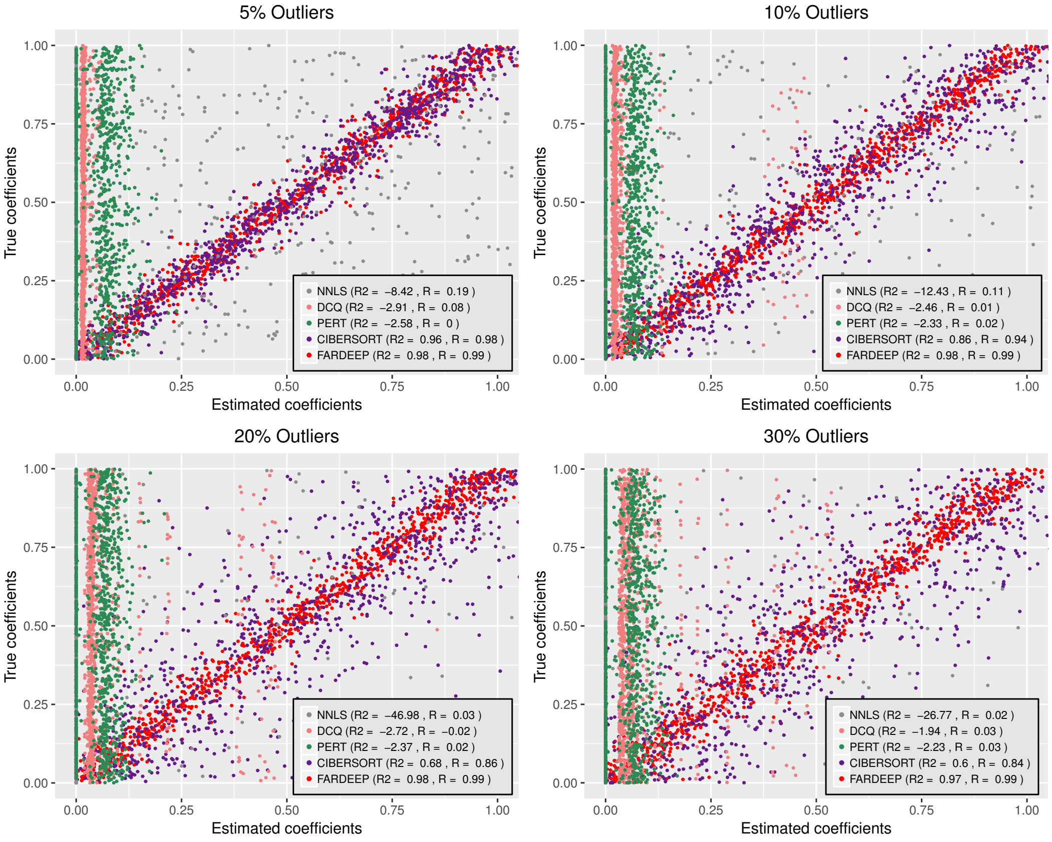

Figure A4: Compare the estimation accuracy of different deconvolution approaches for simulations with correlated random errors (the values in parentheses are R^2 and R). Based on $\{5\%, 10\%, 20\%, 30\%\}$ percentage of outliers, we computed R^2 to evaluate how well are the estimators fit for a straight line $\hat{\boldsymbol{\beta}} = \boldsymbol{\beta}$.
